# Department of Therapeutics

**Published:** 1896-04

**Authors:** 

**Affiliations:** Demonstrator of Physiology and Lecturer on the History of Medicine in the Medical Department of the University of Texas, Galveston


### Current Medical Literature.

### DEPARTMENT OF THERAPEUTICS.

BY DAVID CERNA, M. D., PH. D.
Demonstrator of Physiology and Lecturer on the History of Medicine in the Medical Department of the University of Texas, Galveston.

PECULIAR SYMPTOMS OF TRIONAL POISONING.—An interesting case of poisoning by trional is reported by J. C. Welch, of Bellevue, Pa. (*Medical News*, February 15, 1896). A man 48 years of age, who had been drinking quite heavily, was suffering from insomnia, slight delirium, anorexia and diarrhœa. He was put to bed and ordered thirty grains of trional every three hours, and fifteen minims of the tincture of nux vomica three times a day. The diarrhœa was treated with bismuth and salol. During the following four days the patient slept 2¾, 7½, 17, and 18 hours respectively. The trional was then reduced to 30 grains morning and evening. During the next twenty-four hours he slept seven hours, and during the next day, fourteen hours. He was now noticed to be dull and bewildered, and complained of heaviness and numbness of the limbs, and of great mental depression. His speech was very ataxic; the difficulty of speech was apparently of central origin, and at first a beginning cerebral hemorrhage was feared. In the preceding six days, from two to two and one-half ounce of trional had been taken. The administration of this drug was then entirely suspended, and caffeine and digitalis substituted. All the unfavorable symptoms disappeared, and the insomnia did not return.

---

THE USES OF PHENYLQUINALDINE HYDROCHLORATE.—Phenylquinaldine, C H (C H )N, results from the action of hydrochloric acid upon aniline mixed with acetophenone and paralde hyde, as well as by the condensation of benzoylacetone with aniline. Its hydrochlorate occurs in colorless crystals of a pungent, peppery taste, and readily soluble in water. Applied topically in concentrated solutions, it acts as an irritant. Professor Celli (*Munich. Med. Wochenschr.* 1896. XLII, p. 3), has employed phenylquinaldine hydrochlorate in twelve cases of malaria, in the tentative dose of 0.1 to 0.2 gramme (1¼ to 3 grains), given in wafers. However, Von Ziemssen has found that much larges doses—0.6 to 0.8 gramme (9 to 12 grains)—are usually borne well, occasioning no disturbance; nausea and vomiting are but rarely observed. As far as the reports go, phenylquinaldine hydrochlorate seems to be possessed of some antiperiodic power; but how it compares in this respect with the well-known remedies yet remain to be determined by further clinical observation.—*American Medico-Surgical Bulletin*, February 29, 1896.

---

Treatment of Chorea.—J. Madison Taylor, of Philadelphia (*Philadelphia Polyclinic*, January 11, 1896—*American Medico-Surgical Bulletin*, February 29, 1896) suggests the following therapeutic measures in the systematic treatment of chorea: 1. Specific medication—arsenic—with anti-rheumatic or anti-malarial drugs. 2. Rest to the body, and care to take proper sleep. 3. Nutritional repair—to counteract the depreciation and devitalization of the tissues, caused by their exaggerated overaction. 4. Re-education of co-ordination—a very important but little noticed item. Put the child to bed, warmly clad, with plain food and no red meat, and an abundance of milk, fruit and vegetables. Bath twice daily in tepid water, with spinal douches, followed by brisk rubbing and massage. Give a laxative every second or third day for the first week to get rid of intestinal irritation or fecal toxins. For the anemia cod-liver oil, preferably in capsules, rather than iron. To restore co-ordination, re-educate the limbs by gymnastics and systematic posings (so-called Delsarte exercises), but always insist on rest after the exercises. For rheumatic pain, use salicylates, preferably ammonium salicylate, combined with ammonium bromide in liquor ammonii acetatis or elixir calisaya. Fowler's solution of arsenic gradually increased from three drops thrice daily by the addition of one drop a day until toxic symptoms are produced, is the most satisfactory general method of treatment. Quinine is sometimes of service. If habit chorea develops, moral suasion, hypnotism, or suggestion or mild fright are useful. Reasoning with a child may be efficacious.

---

One Authentic Cure of Leprosy.—Dr. Goldschmidt, of Maderia, describes in the *Bulletin Medical*, December 18, 1895, a case of leprosy cured with europhen is (obutylorthocresoliodide), the only case on record, he believes. The patient showed leprous patches on the face and limbs, which he treated with a solution of 5 grammes pure europhen to 95 grammes olive oil. This was rubbed into the leprous patches with brisk friction twice a day, ten minutes at a time, for four years. It was also applied on cotton at night, with tampons for the nostrils all the time. The leprous patches disappeared, and in some places the skin healed to look normal, in others there are scars, but there is not a trace now of leprosy nor of the leprosy bacillus. The lepers's hospital at Funchal dates from the fifteen century, when convicts and lepers were transported from Portugal, and is the only one of its kind yet in the kingdom. The poverty and insufficient food of the natives favored the development of leprosy, especially as the bath is unknown. Dr. Golschmidt has published a work on leprosy, and has tried every remedy without success, until this case cited above.—*The Journal of the American Medical Association*, February 15, 1896.

---

Resorcin in Skin Disease.—J. Abbott Cantrell, of Philadelphia (*Philadelphia Polyclinic*, February 15, 1896), in an interesting article gives his experience in the use of resorcin in the treatment of cutaneous disorders. The remedy was applied locally: In lentigo the pigment was removed in the majority of cases. In all forms of ringworm, resorcin acted well and generally succeeded in killing the parasite: in tinea circinata, if strong applications were made use of, the remedy was apt to cause a dermatitis; good results were similarly obtained in tinea tonsurans. In acne, and especially in seborrhœa, not only did the drug remove the accumulations, but also acted as a stimulant to the glands by which the quantity and the quality of the proper secretions were improved. The remedy gave good results in dysodrosis and hyperidrosis, and in the latter it prevented both the abnormal secretion and the inflammatory condition of the part. Cases of pytiriasis, capitis and psoriasis improved under the influence of resorcin. The drug produced excellent results in eczema, especially so in eczema rubrum and the squamous varieties, and the same may be said in regard to eczamatous inflammations of the nails. In superficial epitheliomata the drug gave great satisfaction, but failed in extensive deep varieties of the disorder, particularly when the destruction of tissue was considerable. Under resorcin the parasite of impetgo contagiosa lost its power of contagion almost immediately. The effects of the remedy in syphilitic as well as non-syphilitic ulceration, was marvelous; it aided decidedly in the restoration of destroyed tissue, and this action was most marked if during the treatment the patient was confined to bed. The author used resorcin in the following manner: "Solutions in water ranging from 10 to 30 per cent. solutions in collodion of the same strength as above stated; ointments ranging from 10 to 40 per cent. It was found that either petrolatum or lanolin proved the more useful ointment base in cases in which there was not much inflammation, but in those demanding a soothing application, zinc oxide ointment proved more beneficial. Plasters were chosen in cases in which it was impossible to apply ointments or solutions, and their strength varied from 10 to 40 per cent. In cases of acne, in which the drug was applied, it was found more beneficial to make an emulsion with water, adding a small quantity of mucilage of acacia or tragacant, and sometimes a small quantity of oil of rose. This application varied in strength from 5 to 20 per cent., according to the requirements of the case."

